# Structural and quantum chemical basis for OCP-mediated quenching of phycobilisomes

**DOI:** 10.1126/sciadv.adk7535

**Published:** 2024-04-05

**Authors:** Paul V. Sauer, Lorenzo Cupellini, Markus Sutter, Mattia Bondanza, María Agustina Domínguez Martin, Henning Kirst, David Bína, Adrian Fujiet Koh, Abhay Kotecha, Basil J. Greber, Eva Nogales, Tomáš Polívka7, Benedetta Mennucci, Cheryl A. Kerfeld

**Affiliations:** ^1^ California Institute for Quantitative Biosciences (QB3), University of California, Berkeley, CA 94720, USA.; ^2^ Howard Hughes Medical Institute, University of California, Berkeley, CA 94720, USA.; ^3^ Dipartimento di Chimica e Chimica Industriale, Università di Pisa, Via G. Moruzzi 13, I-56124 Pisa, Italy.; ^4^ MSU-DOE Plant Research Laboratory, Michigan State University, East Lansing, MI 48824, USA.; ^5^ Environmental Genomics and Systems Biology Division, Lawrence Berkeley National Laboratory, Berkeley, CA 94720, USA.; ^6^ Molecular Biophysics and Integrated Bioimaging Division, Lawrence Berkeley National Laboratory, Berkeley, CA 94720, USA.; ^7^ Faculty of Science, University of South Bohemia, Ceske Budejovice, Czech Republic.; ^8^ Biology Centre of the Czech Academy of Sciences, Ceske Budejovice, Czech Republic.; ^9^ Thermo Fisher Scientific, Eindhoven, Netherlands.; ^10^ Division of Structural Biology, The Institute of Cancer Research, London SW7 3RP, UK.; ^11^ Department of Molecular and Cellular Biology, University of California, Berkeley, CA 94720, USA.; ^12^ Department of Biochemistry and Molecular Biology, Michigan State University, East Lansing, MI 48824, USA.

## Abstract

Cyanobacteria use large antenna complexes called phycobilisomes (PBSs) for light harvesting. However, intense light triggers non-photochemical quenching, where the orange carotenoid protein (OCP) binds to PBS, dissipating excess energy as heat. The mechanism of efficiently transferring energy from phycocyanobilins in PBS to canthaxanthin in OCP remains insufficiently understood. Using cryo–electron microscopy, we unveiled the OCP-PBS complex structure at 1.6- to 2.1-angstrom resolution, showcasing its inherent flexibility. Using multiscale quantum chemistry, we disclosed the quenching mechanism. Identifying key protein residues, we clarified how canthaxanthin’s transition dipole moment in its lowest-energy dark state becomes large enough for efficient energy transfer from phycocyanobilins. Our energy transfer model offers a detailed understanding of the atomic determinants of light harvesting regulation and antenna architecture in cyanobacteria.

## Teaser

High-resolution cryo-EM structure of OCP-PBS reveals intrinsic motions and enables atomic simulation of the quenching mechanism.

## INTRODUCTION

In photosynthetic organisms, light-harvesting antennae capture and direct energy through elaborate networks of protein-scaffolded pigments to ultimately be used to fix CO_2_. In cyanobacteria, the principal antenna complex is the phycobilisome (PBS). In these massive macromolecular assemblies, light-absorbing phycocyanobilin (PCB) pigments are covalently bound to phycobiliproteins that are tethered together by linker proteins and organized into the PBS rods and core cylinders. The protein environment of the pigments tunes their spectral properties, allowing for efficient and fast transfer of the excitation energy toward the photosynthetic reaction centers.

The efficiency of photosynthesis relies on balancing the delivery of excitation energy and the ability to photoprotect under conditions in which the harvested light energy exceeds photosynthetic capacity. Non-photochemical quenching (NPQ) is the process by which excess captured light energy is dissipated as heat (*1*). In cyanobacteria, NPQ is carried out by the orange carotenoid protein (OCP), a 34-kDa protein containing a single keto-carotenoid (*2*, *3*). It converts from a resting, orange form (OCP^O^) to a red, active form (OCP^R^) after absorption of intense blue light. Two OCP^R^ homodimers then bind to the PBS core, activating quenching (*4*). Although it is indisputable that OCP causes quenching in the PBS, the quenching mechanism itself is still debated (*2*). Excitation energy transfer (EET) from the PBS PCB to the OCP-bound keto-carotenoid appears as a promising explanation. Our recent structure of the OCP-PBS complex (*4*) supports this picture, as the keto-carotenoid [canthaxanthin (CAN)] is located close to two ApcA PCBs. However, EET is only efficient if there is resonance between the donor (PCB) emission and acceptor (CAN) absorption and if there is a sizable electronic coupling between the two electronic transitions, i.e., if the corresponding transition dipole moments (TDMs) are large (*5*). CAN, like all carotenoids, has two low-energy singlet excited states, S_1_ and S_2_, with different transition properties. The S_0_ → S_1_ transition cannot be induced by a one-photon absorption because of the very small TDM, which makes S_1_ optically dark and an inefficient EET acceptor although its energy is in resonance with the PCB emission. Conversely, the second excited state, S_2_, is optically bright and responsible for the intense absorption of carotenoids, but its energy is too high to allow EET from PCB. A somewhat large TDM for the S_1_ state had to be assumed to match the quenching rates observed experimentally (*4*). However, it is not clear how this large TDM is achieved in OCP-bound CAN.

Recent developments in cryo–electron microscopy (cryo-EM) data acquisition and processing have made it possible to obtain structures of well-behaved test specimens at near-atomic resolution (≤2 Å), with the promise to extend this resolution goal to more challenging targets in physiologically relevant states (*6*, *7*). More accurate atomic models obtained from such high-resolution structures are bringing insight into the role of local intrinsic protein motions in large macromolecular assemblies and are useful to determine properties of quantum mechanical processes such as pigment excitation that is especially relevant in photosynthetic protein complexes (*8*, *9*).

We were previously successful in obtaining cryo-EM structures of quenched and unquenched PBS from the model cyanobacterium *Synechocystis* sp. PCC 6803, but our efforts stopped short of offering a detailed model of energy transfer that considers the local protein environment and positions of associated water molecules (*4*). Such a model is necessary to understand in detail how a small protein with a single carotenoid can fully quench the 6.2-MDa PBS.

Here, we take advantage of the latest cryo-EM developments to determine the structure of the quenched PBS bound to OCP to a resolution of 2.1 Å in the core and 1.8 Å in the rods, with some regions reaching 1.6-Å resolution. This enabled building of an unprecedentedly accurate model of the structure, including ordered water molecules and hydrogen atoms, and the characterization of local intrinsic motions within the PBS. We then used this atomic model to perform multiscale quantum chemical calculations that reveal the interplay between pigments and protein residues, enabling OCP to serve as an energy sink for the PBS. We identify several residues within OCP that are critical to establish the sizable TDM along CAN that is required for quenching. The energy transfer model derived from our structure explains the fast EET from the ApcA PCBs to CAN and provides a better understanding of the atomic determinants of light harvesting and energy transfer and its regulation, the latter including revealing macromolecular motions within the PBS.

## RESULTS

### High-resolution cryo-EM structure of OCP-PBS

**Fig. 1.**
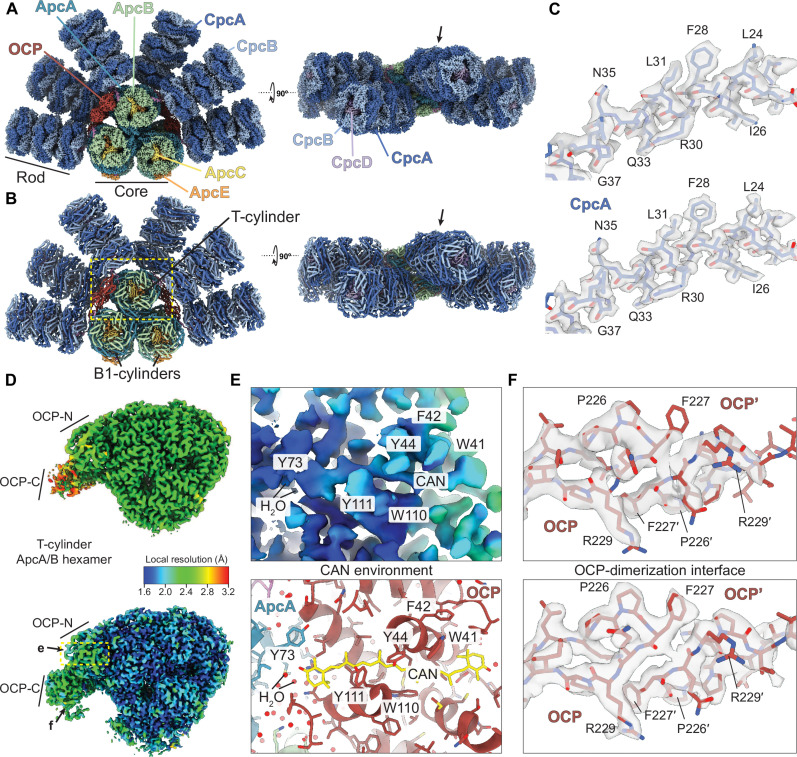
Structure of the OCP-PBS. (**A**) Composite cryo-EM map of the OCP-PBS colored by subunit type. Arrow points to molecular detail shown in (C). (**B**) Cartoon representation of the OCP-PBS atomic model with the arrow pointing to molecular detail shown in (C). Yellow dashed box marks the region of the OCP-PBS model shown in (D). (**C**) Map details for the rod in previous (top) and new (bottom) cryo-EM reconstructions. (**D**) Local resolution map of the T-cylinder disk bound to OCP in our previous reconstruction (top) compared to our new reconstruction (bottom). Arrows indicate the location of details shown in (E) and (F). (**E**) Local resolution within the N-terminal domain (NTD) of OCP surrounding the CAN (top) and corresponding atomic model (bottom). (**F**) Map quality of the OCP dimerization interface of our previous reconstruction (top) compared to this study (bottom).

To obtain a more detailed understanding of OCP-mediated PBS quenching, we sought to determine a high-resolution cryo-EM structure of the OCP-PBS complex, taking advantage of recent technological developments in data acquisition and processing. We used our previously established protocol for streptavidin affinity grids to stabilize the sample on the cryo-EM grid (*10*, *11*) and then acquired the data on a 300-kV cryo-electron microscope equipped with a Falcon 4 direct electron detector with increased detective quantum efficiency (DQE) at Nyquist frequency, a cold field emission gun, and an energy filter capable of maintaining a narrow slit width for extended periods of time (*6*, *12*). This setup allowed us to maximize the signal-to-noise ratio as compared to conventional microscopes, particularly at resolutions beyond 2 Å. To further increase the resolution and to combat loss of data quality caused by protein complex flexibility intrinsic to all biological macromolecules, we performed three-dimensional (3D) variability and 3D flexibility analysis as implemented in recent software packages (*13*, *14*). Using these approaches, we were able to obtain a structure of the OCP-PBS complex from *Synechocystis* sp. PCC 6803 with a resolution of up to 2 Å in the core and 1.8 Å in the rods (see Materials and Methods), an improvement of ~0.3 to 0.5 Å for the rods and the core, respectively (Fig. 1, A and B, figs. S1 and S2, and table S1). The newly obtained reconstruction recapitulates the known OCP-PBS structure, consisting of a tri-cylindrical core composed of Apc proteins with six emanating rods composed of Cpc proteins. On each side of the core, one OCP dimer is wedged between the top and bottom cylinders, as previously reported. Within one OCP dimer, the two OCPs connect via their C-terminal domain (CTD), while their N-terminal domains (NTDs) and their embedded CAN molecules bind to AcpA and ApcB of the PBS core. The gain in resolution allowed us to build an atomic model of the OCP-PBS with increased accuracy compared to our previous structure (Fig. 1B). High-resolution details were readily visible in several parts of the map, for example, in the rods, where characteristic holes in the density of aromatic residues became apparent (Fig. 1C). Within the NTD of the OCP, the local resolution of CAN and its surrounding residues has increased to 1.7 to 2 Å, and, therefore, the density for all four methyl moieties branching off the polyene chain and for the two terminal beta-ionone rings can be clearly assigned (Fig. 1, D and E). The new map also shows more continuous density for the CTD of OCP and the quality of the map for the CTD increased further after accounting for protein flexibility. Regions of OCP-CTD that were previously poorly resolved, especially around the CTD dimerization interface, now show clear continuity and side-chain density, confirming the proposed interface (Fig. 1F).

In addition, we were able to model more than 7000 unique water molecules that were previously not visible, including 49 water molecules at the OCP-PBS interface (Fig. 1E), allowing us to include them into our energy transfer and quenching calculations (see below). In addition, we can identify density for the posttranslational modification of residue Asn^72^ of CpcB to N4-methyl-asparagine (fig. S3) (*15*, *16*).

Analysis of the data using ResLog indicated that resolution of our maps was limited mainly by particle numbers and not by sample heterogeneity (fig. S4A) (*17*). Therefore, to explore to which extent resolution detail can be improved, we took advantage of the local D3 symmetry within the central Cpc(αβ)_6_ double hexamer of the rod (“central rod disk”) to improve the signal-to-noise ratio. Applying D3 symmetry resulted in a local map of the central rod disk with a resolution of 1.6 Å, with the details expected at this resolution clearly visible (fig. S4, B to D). The same particles yielded a resolution of 1.8 Å when not applying any symmetry (C1). Calculating difference maps allowed us to visualize hydrogen density along the c-α backbone of several α helices (fig. S4D). We believe that the use of streptavidin affinity grids was particularly critical to get significantly higher resolution than any other published PBS structure so far. This type of grid greatly alleviated the issue of preferential orientation of the PBS and protected the particles from harmful interactions with non-biological interfaces, such the air-water interface.

**Fig. 2.**
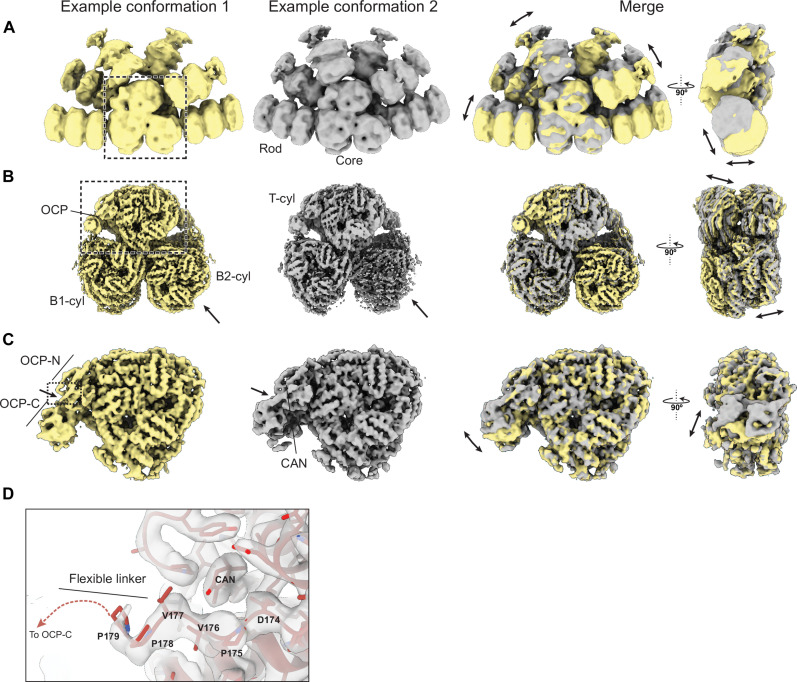
Visualization of distinct forms of motion in OCP-PBS at different scales. (**A** to **C**) The two extreme states within a certain motion are displayed next to each other (yellow and gray) and the superimposed and displayed in two orthogonal views. (A) Holo–OCP-PBS. The double arrows indicate the direction of rod movement. (B) OCP-PBS core. The arrow points to the position of loss of ApcA/B double hexamer in the B2-cylinder and the double arrows indicate the direction of twisting movement. (C) T-cylinder disk bound to OCP. The single arrow points to the location of the flexible linker between the NTD and CTD of OCP and the double arrows show the direction of OCP-CTD movement. The dashed box shows the part of the structure that is enlarged in (D). (**D**) Molecular details around the flexible linker of OCP. The linker residues proximal to the CAN molecule are indicated.

Previous cryo-EM reconstructions of the cyanobacterial PBS suffered from inherent flexibility of the complex that made rods appear “smeared out” or incomplete (*4*). The high quality of our data and recent software developments allowed us both to increase the overall map quality of several domains of the PBS (Fig. 1, C to E) and to extract biologically relevant information about conformational variability. Using 3DVA and 3DFlex (*13*, *14*), we investigated the intrinsic movement on three scales: the entire OCP-PBS, its tri-cylindrical core, and the T-cylinder bound to OCP (Fig. 2). The overall movement of the holo–OCP-PBS complex is dominated by movement of the six rods that emanate from the core region (Fig. 2A and movie S1). The rods rock “up” and “down” in small angles as rigid units relative to the core. The overall displacements are in the range of 20 Å, and they are less pronounced in the “forth” and “back” directions. We previously proposed that the rods in the PBS are mobile and that they can adopt distinct conformations that control access of OCP to the PBS core (*4*). Our flexibility analysis shows that the rods have an inherent propensity to move, in agreement with that hypothesis. We anticipate that within the cell the intrinsic mobility will have an impact on the supramolecular assembly and disassembly of PBS arrays to regulate light harvesting.

The core of the OCP-PBS complex displays an overall slight twisting motion, with the top cylinder and bottom cylinders moving in opposite directions around the central symmetry axis (Fig. 2B and movie S2). Our analysis also revealed that one of the B-cylinder’s ApcA/B hexamers is lost in a subset of particles. While this could occur during cryo-EM sample preparation, it agrees with previously reported sample instability or heterogeneity and may give rise to the presence of spectroscopically active components in PBS samples that are distinct from the holo-complex (*18*, *19*).

Within the isolated T-cylinder disk bound to OCP, structural flexibility appears mostly restricted to the motion of the dimerized CTDs of OCP with respect to the remainder of the complex (Fig. 2C and movie S3). The most prominent motion of the dimerized CTDs of OCP appears to be an up and down rocking relative to the core. The flexible linker connecting the NTD and CTD of OCP moves in concert with the CTD dimer. Residues D174-V176 within the linker are in close proximity to the CAN molecule that is embedded in the NTD of OCP^R^ (Fig. 2D). Because the protein environment of CAN affects its electronic properties and, therefore, its ability to perform NPQ, we asked whether the intrinsic flexibility of OCP would play a role in regulating this effect. To answer this question, we used our new atomic model of the OCP-PBS to calculate the excited-state parameters of CAN and to determine the mechanism of CAN-mediated NPQ in detail.

### Multiscale quantum chemical calculations on OCP-PBS

**Fig. 3.**
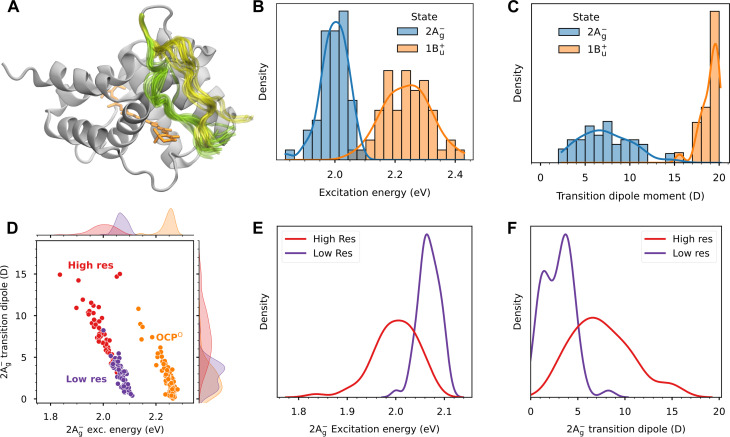
Linker flexibility and CAN excited-state properties. (**A**) Flexibility of the linker in our restMD simulations. Representation of the NTD in OCP-PBS (atoms of the PBS are omitted) along two restMD replicas. The colored parts (yellow and green for the two replicas) correspond to the linker residues G169-P179 that were allowed to move during the MD. CAN is shown in orange. (**B** and **C**) Distribution of the excitation energy (B) and TDM (C), for the dark (2A_g_^−^) state and the bright (1B_u_^+^) state calculated along the restMD trajectories. (**D**) Comparison of dark-state properties in OCP-PBS and OCP^O^. Points represent pairs of excitation energy and TDM for each structure sampled from OCP-PBS (orange) or OCP-PBS (red), whereas the shaded curves on the top and right sides represent the marginal distribution of excitation energy (top) and TDM (right). Points corresponding to the previous lower-resolution OCP-PBS structure (Low res) (*4*) are shown in purple. (**E** and **F**) Comparison between CAN S_1_ properties obtained with QM/MM calculations on restMD simulations from high-resolution structure (High res) in this study and from the previous lower resolution structure (Low res). (E) Excitation energy and (F) TDM of the S_0_-S_1_ transition.

Our previous model describing the energy transfer quenching of PBS by OCP (*4*) used empirical values for two key parameters of the carotenoid: the TDM of CAN in OCP and its S_0_-S_1_ transition energy. Here, in contrast, we exploit our higher-resolution structure to apply quantum mechanics/molecular mechanics (QM/MM) calculations in addition to restrained molecular dynamics (restMD) simulations to obtain more realistic values of these parameters accounting for the effects of CAN-protein interactions (see Materials and Methods). Given the large size of the entire PBS core, in our OCP-PBS model, we considered only the closest regions of the core to the OCP-NTD, which is bound to the T-cylinder of PBS. To sample solvent configurations, we performed restMD simulations in explicit water solvent where the system was restrained on the backbone of the proteins of our high-confidence model. Sampling of water configurations is essential to accurately determine the effects of the solvent on the electronic structure of CAN. It has been observed that bulk water can partially screen the protein’s electrostatics in both OCP^O^ and NTD (*20*). As evident from cryo-EM, the OCP-CTD and its linker region are inherently flexible, which could tune the electronic properties of CAN. To investigate this hypothesis, the linker was left unrestrained in the restMD simulations to fully sample its configurational space. Because of the large distance between CAN and the CTD, the latter was excluded from the model. In agreement with the structural data, our restMD simulations show that the linker exhibits substantial flexibility (Fig. 3A) and that it can acquire different conformations and positions. Furthermore, the regions of high water occupancy in the restMD simulations mirror the positions of resolved water molecules in the cryo-EM structure (fig. S6) and the positions of CAN and the closest polar residues sampled by restMD fit well into the cryo-EM map (fig. S9).

#### Excited-state properties of CAN

As commented in Introduction, the photophysics of carotenoids is commonly described by a three-level model: the ground state (S_0_) and the two lowest excited states, the dark S_1_ (generally indicated in terms of its ideal symmetry, $2A_{g}^{-}$ ), and the bright S_2_ (with ideal $1B_{u}^{+}$ symmetry). Following this model, we extracted structures for the OCP-PBS model from two independent restMD trajectories and calculated the excitation energies and the corresponding TDMs for the S_0_-S_1_ and S_0_-S_2_ transitions of CAN using a semiempirical QM method (*21*), coupled to a MM description of the protein matrix and the solvent. We find that, in agreement with earlier hypotheses (*4*), the S_0_-S_1_ TDM of CAN in OCP-PBS is substantially increased with respect to the isolated CAN (Table 1), although it remains always significantly lower than the one found for the S_0_-S_2_ bright transition (Fig. 3, B and C). Notably, we observe a broad distribution of TDM values for the dark state, sometimes exceeding 10 D, and with an average (7 D) much larger than the 2.3 D obtained from previous calculations on echinenone in OCP^O^ (*22*). As in our restMD the well-resolved regions of the protein are restrained, together with the CAN, the TDM distribution only arises from the dynamics of water and external side chains, which modulate the protein electric field acting on the embedded carotenoid.

**Table 1.** Excitation energies (Δ*E* in electron volts) and TDMs (in debye) of the first two electronic transitions of CAN calculated using different geometries and environments.

	S_0_-S_1_ (2A_g_^−^)		S_0_-S_2_ (1B_u_^+^)	
	Δ*E*	TDM	Δ*E*	TDM
CAN in gas phase (gas-phase geometry)	2.74	<0.1	2.85	17.4
CAN in gas phase (OCP-PBS geometry)	2.13	0.3	2.57	17.4
CAN in OCP-PBS (MD average)	2.00	7.8*	2.23	19.0*
CAN in gas phase (OCP^O^ geometry)	2.28	0.6	2.64	18.6
CAN in OCP^O^ (MD average)	2.24	3.3*	2.53	18.4*

*The TDM average was obtained as root mean square of the TDM vectors calculated along the restMD trajectories.

To assess the impact of the flexible linker on the properties of CAN, we performed an additional MD simulation excluding the OCP linker residues G169-P179. In this model, the calculated S_0_-S_1_ TDMs are slightly smaller, while the corresponding transition energies do not differ significantly from the ones with the linker (fig. S7). Therefore, the flexibility of the linker only has a minor effect on the transition properties of CAN.

To quantitatively assess the importance of a well-resolved structure around the CAN, we repeated the same restMD and QM/MM calculations on the OCP-PBS complex, using the previous structure restricted to a resolution of 2.6 Å (*4*). This analysis allowed a direct comparison of the dark state properties (Fig. 3, D to F). The previous structure gave significantly smaller TDMs and higher excitation energies than the structure from our present work. Specifically, the earlier structure of OCP-PBS did not give significantly different TDMs relative to OCP^O^, whereas the TDMs obtained on the present structure are much larger (Fig. 3D).

The comparison with OCP^O^ also shows that the S_0_-S_1_ transition energy is redshifted by 0.2 eV in OCP-PBS, with a somewhat broader distribution. Furthermore, TDMs in OCP-PBS are substantially larger than in OCP^O^. The average value of S_0_-S_1_ TDM in OCP^O^ is around 3 D and its distribution is centered between 1 and 2 D, which is close to the earlier calculations (*22*). This comparison suggests that the specific electrostatic environment of CAN in OCP-PBS substantially increases the TDM of the S_0_-S_1_ transition. Looking at results calculated using a geometry of CAN optimized in gas-phase and the ones imposed by the OCP-PBS complex and the OCP^O^, respectively (Table 1), it is clear that the differences in the properties of CAN cannot be explained only in terms of a geometrical effect. A similar behavior is found for the overall absorption spectrum of CAN in OCP-PBS and OCP^O^ (fig. S10), in agreement with experiments (*23*, *24*).

**Fig. 4.**
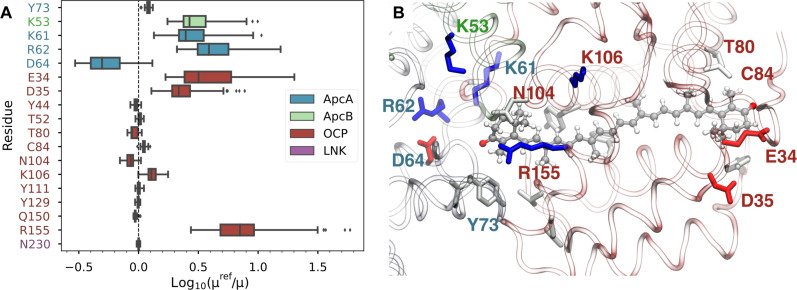
Impact of the molecular environment on the CAN S_1_ TDM. (**A**) Effect of selected residues on the TDM of CAN (S_1_ state), computed as the logarithm of the ratio between the full OCP-PBS calculation (μ^ref^) and the calculation excluding the selected side chain (μ). (**B**) Representation of the most important residues in the CAN binding pocket. Colors indicate the residue type: blue for Lys/Arg, red for Asp/Glu, and gray/white for polar/nonpolar residues. Label colors refer to each side chain with the same color code as in (A).

The analysis of QM/MM simulations shows that the significant TDM of the dark state of CAN in OCP-PBS is mainly induced by a marked imbalance of charge distribution within the CAN binding pocket. By analyzing the effect of each residue separately (see Materials and Methods), we found that almost all charged residues close to CAN, in OCP or in the PBS, contribute to the increase of the TDM (Fig. 4). Proximal to the PBS, R155 in OCP has the largest effect on the TDM, followed by some positively charged residues on ApcA (K61 and K62) and ApcB (K53). At the same time, the negatively charged residues, E34 and D35, which are located on the distal, solvent-exposed surface of OCP, also increase the TDM. The only charged residue that decreases the TDM is D64 on ApcA, which is needed to make a salt bridge with R62. Thus, it appears that the positive and negative residues on either end of the CAN give rise to an increase in the S_0_-S_1_ TDM by generating a strong electrostatic potential difference that breaks the symmetry along the conjugated chain of the carotenoid. Several highly conserved residues of the PBS located at the OCP binding interface (ApcA-K61, ApcA-R62, and ApcB-K53) contribute to the TDM increase, suggesting that OCP-PBS binding is essential to achieve a large TDM in the CAN. Notably, removing any of the charged residues at either end of the CAN results in at least threefold decrease of the TDM. Although this estimate only covers the direct electrostatic effect of the residues (see Materials and Methods), this analysis strongly suggests that only in the OCP-PBS complex the dark state of CAN can acquire a large TDM.

Our calculations also indicate that the distortion of the carotenoid in OCP-PBS is not sufficient to achieve a significant TDM for the S_0_-S_1_ transition. Instead, the charges of the protein environment substantially increase the S_0_-S_1_ TDM by creating an electrostatic gradient along the CAN-conjugated chain. The TDM increases by virtue of a mixing between the states: The S_1_ state borrows dipole strength from the S_2_ state (fig. S5). Our calculations show that the S_1_ and S_2_ states are close in energy, at least at the Franck-Condon point (Fig. 3B), and the increase in S_0_-S_1_ TDM is only substantial when there is a small energy difference between S_1_ and S_2_ (fig. S5C). We lastly note that, even if such an increased TDM (∼8 D) should be visible in the absorption spectrum, our simulations show that the corresponding band is hidden in the red edge of the more intense S_0_-S_2_ band (fig. S5D).

#### Energy transfer and OCP-mediated quenching

**Fig. 5.**
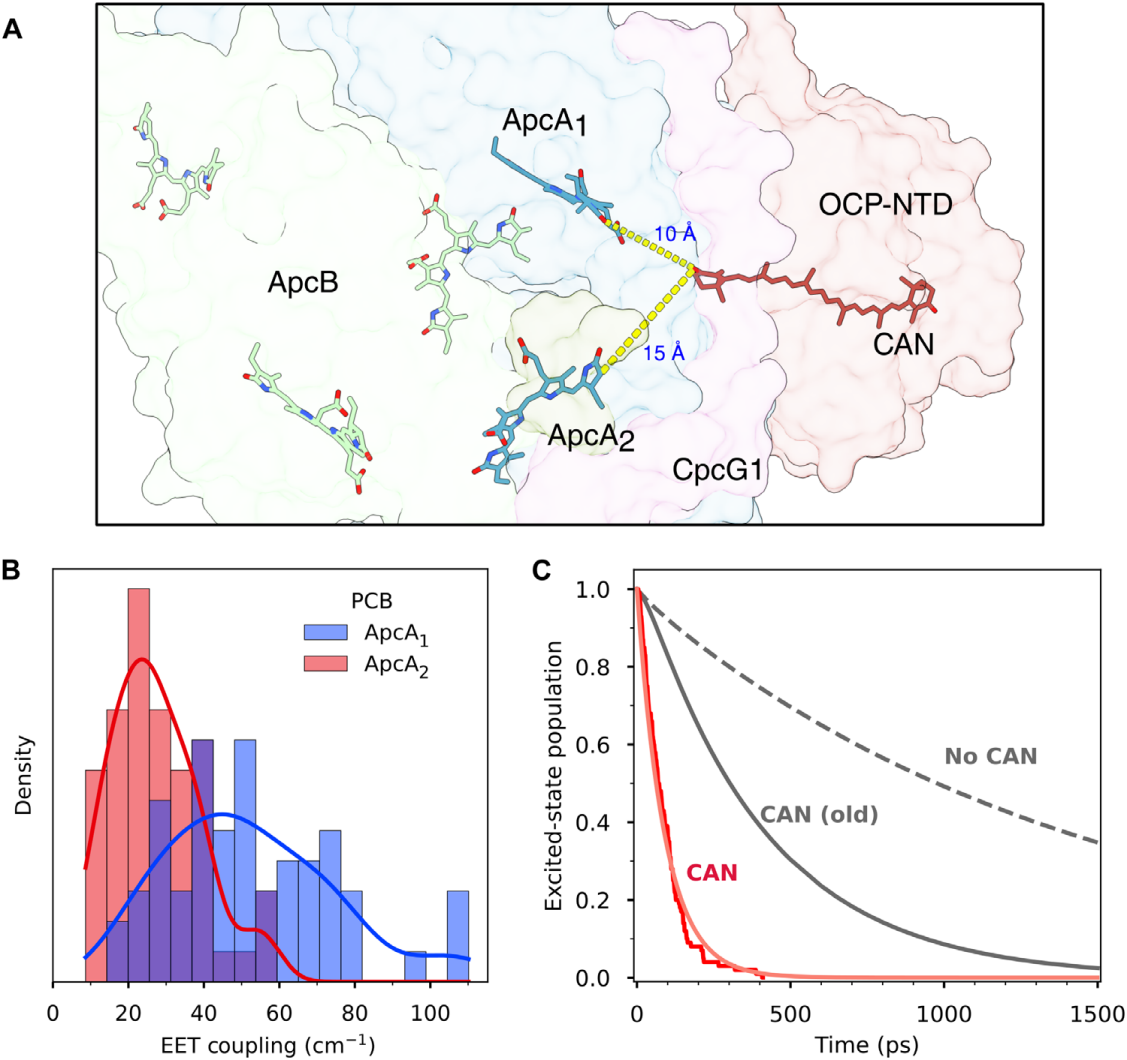
Simulation of EET quenching and the excited-state decay in the OCP-PBS complex. (**A**) Representation of CAN in OCP and the closest Apc pigments. The closest ApcA pigments are denoted as ApcA_1_ and ApcA_2_. (**B**) Distribution of the calculated EET couplings between CAN S_0_-S_1_ and ApcA1/2. (**C**) Results of 100 stochastic simulations of PBS decay in the presence of four CAN parametrized with QM/MM simulation results (red), excited-state decay simulation from (*4*) for OCP-PBS quenched by four CAN (gray solid line), unquenched PBS (dashed gray line).

The same QM/MM calculations along the restMD trajectories were used to compute EET couplings of CAN with the closest PCB pigments. The closest PCBs are those of two different ApcA subunits; one of them is only 10 to 15 Å away from the CAN (Fig. 5A). Therefore, the point-dipole approximation is not fully justified here, and we used the transition charges from electrostatic potential (TrEsp) method (*25*) for calculating the CAN(S_1_)-PCB couplings (see Materials and Methods). The coupling distributions (Fig. 5B) are quite broad and reflect the observed variability in the TDM. Although we have not used the TDM for computing the couplings, both CAN-ApcA couplings are essentially proportional to the TDM of CAN (fig. S8). This result is expected because both the TrEsp charges and the TDM are proportional to the mixing between the S_1_ and S_2_ states. The mean couplings, 54 cm^−1^ for ApcA_1_ and 27 cm^−1^ for ApcA_2_, are compatible with significant PCB-to-CAN EET rates. The other (ApcB) pigments have couplings smaller than 8 cm^−1^.

We used the CAN(S_1_)/PCB couplings to calculate the quenching of PBS by CAN in OCP. Simulation of the EET dynamics was performed essentially as described by Dominguez-Martín *et al.* (*4*). Here, however, the point-dipole approximation was used only for the PCB-PCB couplings within PBS to model the energy flow through PBS, whereas the individual couplings between CAN and the two nearest ApcA PCBs were directly taken from the QM/MM TrEsp calculations. The other CAN-PCB couplings were neglected because our previous analysis showed that their contribution to the quenching is negligible (*4*). The CAN(S_1_)-PCB(ApcA) couplings and the S_0_-S_1_ energies of CAN were randomly sampled from the QM/MM distributions shown in Fig. 5B and Fig. 3B and assigned independently to each of the four OCP carotenoids.

We have used the same estimated S_0_-S_1_ absorption band shape used in (*4*) but with distribution of absorption maximum according to Fig. 3B. The simulation was then run using the stochastic approach, starting from a randomly selected rod PCB. For each run, the CAN parameters were independently sampled. The results of simulations are shown in Fig. 5C. The effect of the increased CAN-PCB coupling due to large S_0_-S_1_ TDM of CAN in OCP-PBS is evident. The overall PBS lifetime drops to ∼100 ps providing all four OCPs are attached to PBS, in agreement with a lifetime of ∼160 ps, which has been determined experimentally (*18*).

## DISCUSSION

We have presented the high-resolution cryo-EM structure of a cyanobacterial PBS in complex with OCP and characterized its inherent flexibility. Some degree of flexibility is inherent in all protein complexes, but how flexibility affects protein function is only beginning to be understood. The stochastic sampling of small conformational changes can immediately translate to changes in protein activity, as has been shown for HIV protease or triose-phosphate isomerase (*26*–*29*). Ensemble averaging techniques like NMR or computational modeling can be used to describe such motions, but these methods are still limited in the size of the system that can be investigated. On the other hand, a common drawback of cryo-EM structural studies is that they suffer from insufficient resolution. Hence, only a few complex dynamic systems, such as the translating ribosome, have been successfully investigated in detail (*30*–*32*). Continued progress in cryo-EM sample preparation and data processing holds unique promise for future studies of dynamic protein complexes, by improving the achievable resolution and allowing more sophisticated analysis of the inherent motion within the dataset.

In examining the OCP-PBS complex, we identified several movements with potential implications for its light-harvesting function. In particular, the inherent movement of the PBS rods hints at a conformational switching that controls access of OCP binding to PBS (*4*). Other regions of the PBS, such as the top cylinder and the NTD of the OCP, are stable. In contrast, there is considerable flexibility in the CTD dimer and the NTD-CTD linker, potentially affecting stability or longevity of the quenched PBS. The role of the observed intrinsic motions in regulating light harvesting can now be tested.

The substantially increased accuracy achieved in our atomic model allowed us to perform a detailed QM/MM simulation of EET quenching of PBS by OCP. Our results predict a substantially larger S_0_-S_1_ TDM for CAN in OCP-PBS than previously estimated (*22*). This leads to larger EET couplings of CAN with the closest ApcA PCB (Fig. 3D), making the OCP an efficient quencher. The simulation results (Fig. 5C) show that tuning the excited-state properties of CAN by the environment in OCP-PBS significantly enhances the quenching rate. While in our previous simulation (*4*), using parameters estimated from other systems, we obtained a 300- to 400-ps time constant for APC-to-CAN energy transfer, by using the QM/MM calculations on the real system, the quenching time decreases to ∼100 ps (Fig. 5C), which corresponds well to the value obtained from time-resolved fluorescence experiments (*18*). This clearly demonstrates the importance of CAN-protein interactions in tuning the quenching because they enhance the quenching rate nearly fourfold compared to that calculated earlier without involving the effect of these interactions (Fig. 5C). The variability observed along our restMD trajectories in EET couplings (and consequently in EET rates) not only suggests that the efficiency of EET quenching is highly tunable by the protein environment but also explains the heterogeneity observed in quenched PBS (*33*). We also note that the APC-to-CAN quenching pathway has been recently observed experimentally using femtosecond transient absorption spectroscopy (*34*). The quenching rates obtained from experiment are close to those obtained here, although Liguori *et al.* assigns the quencher to the heavily-debated S* state of the carotenoid (*35*, *36*).

Comparing our simulations on the high-resolution structure with the previous one at lower resolution, we showed that the 2.6-Å resolution in the previous structure could not provide qualitatively correct results in the QM/MM calculations. Given the extreme sensitivity of the CAN electronic structure to the electrostatic environment, even small differences in the structure could reflect markedly on the QM/MM results. In particular, we can observe a different position of R155 (fig. S11) within the OCP NTD, which is located close to the CAN end and has a substantial effect on its excited-state properties (Fig. 4). Furthermore, other differences can be found in the charged residues of ApcA in the PBS. Overall, the improved structural resolution allowed us to better pinpoint the position of charged side chains, which ultimately determines the success of our QM/MM strategy.

The calculations using the high-resolution structure confirm that, to achieve an efficient quenching, there is no need to quench the lowest energy state emitting at 680 nm, which is associated with ApcD and ApcE subunits (*37*, *38*). Instead, quenching of ApcA pigments emitting at 660 nm is enough to provide photoprotection, as suggested in earlier reports based on analyses of time resolved fluorescence data (*39*, *40*). Moreover, interaction of OCP with ApcA provides a natural way for switching between the quenched and non-quenched state of the PBS. First, changing the position of the rods can prevent OCP binding by blocking the binding site (*4*), thus keeping PBS in an unquenched state. Second, when OCP is bound, the binding site can still allow OCP to interact with FRP, which is needed to revert to inactive OCP^O^. Such regulation would be complicated (if not impossible) to achieve if the quenching site was associated with the lowest energy state, because of the limited access to the ApcE and ApcD subunits (*4*).

In summary, our study illuminates structural dynamics and energy transfer quenching processes within the cyanobacterial PBS through high-resolution cryo-EM and multiscale quantum chemical calculations. These details open avenues for fundamental research on energy transfer in pigment-protein complexes. Likewise, this high-resolution picture of the PBS and its mechanism of NPQ serve as inspiration for synthetic biologists, chemists, and materials scientists to design sustainable technologies for harnessing the clean and abundant energy in sunlight.

## MATERIALS AND METHODS

### High-resolution cryo-EM data acquisition and processing

Plunge-frozen grids prepared and imaged previously (*4*) were provided to Thermo Fisher Scientific, Eindhoven, The Netherlands, for data collection. A total of 16,284 exposures were collected on a Krios G3i microscope operated at 300 kV and equipped with a cold field emission electron gun, Selectris X energy filter, and Falcon 4 camera. Exposures were collected with a nominal pixel size of 0.727 Å and recorded in EER file format with 1176 total raw frames per movie using aberration free image shifting and fringe-free imaging (*6*, *41*). The defocus range was between −0.4 and −1.2 μm, and the total dose per exposure was 40 electrons/Å^2^.

Images were motion-corrected using motioncor2 with variable frame grouping optimized for early frames with high beam induced motion (*42*). During motion correction, frames were temporarily upscaled from 4 to 8 K to improve signal-to-noise at high resolutions. After motion correction, the streptavidin lattice was subtracted using in-house scripts (*10*, *11*). For subsequent processing motion-corrected and subtracted micrographs were imported into cryoSPARC v.4.2 (*10*, *11*, *43*). After CTF estimation, PBS particles were picked using our previous structure as a template. After several rounds of 2D and 3D classification, as well as global and local CTF refinement, we obtained a single class corresponding to OCP-PBS at a global resolution of 2.2 Å according to the Fourier shell correlation (FSC) of 0.143 criterion (*44*). To analyze flexibility of the holo–OCP-PBS, we performed 3D variability analysis as implemented in cryoSPARC, filtering the map to 20 Å and using three modes (*13*).

To obtain a better map of the central core of OCP-PBS, in lieu of local refinements, particles centered on coordinates for holo-complexes were re-extracted at smaller box sizes, effectively truncating the peripheral rods. The map was refined to 2.2 Å and used for subsequent flexibility analysis and local refinements. For flexibility analysis, we ran 3D variability analysis with a 4-Å filter and three modes (*14*).

After symmetry expansion, local refinement of the T-disk yielded a 2.1-Å map, which was used as a basis for 3DFlex analysis as implemented in cryoSPARC, which we ran using five latent dimensions and 307,000 particles.

To obtain reconstructions of the rods, templates from our previous structure were used to pick and extract rod particles in a separate workflow from holo-PBS particles. After 2D classification, 3D classification, and global and local CTF refinement, we obtained a final map at 1.9-Å resolution. Local refinement of the central rod disk yielded a reconstruction at 1.8 Å. Applying D3 symmetry then yielded a reconstruction at 1.6 Å, although it lacked the asymmetric central linker protein portion.

### Atomic modeling and hydrogen modeling

Atomic models were built by first fitting the respective existing structures or subsets of them [Protein Data Bank (PDB) IDs 7SCC, 7SCB, and 7SCA] into the cryo-EM density using UCSF ChimeraX 1.3 (*45*). Hydrogen atoms were added using phenix.reduce (*46*), and a morph step was performed during the initial refinement with phenix.refine 1.19.2 to fit the model into the density. Manual rebuilding with COOT 0.97 (*47*) was alternated with refinement with phenix.refine 1.19.2. Water molecules were added for the final rounds of refinement using phenix.douse and manually evaluated by inspection in COOT 0.97. To visualize the signal from hydrogen atoms in the 1.6- and 1.8-Å maps of the central rod disk, we used the program Servalcat as implemented in Refmac5 and ccpEM v. 1.6.0. to refine the atomic model and create *F*_o_-*F*_c_ maps (*48*). Structures were visualized with UCSF ChimeraX (*45*).

### Molecular dynamics

System preparation and all MD simulations were performed using Amber 18 (*49*). The model system (OCP-NTD and the seven closest apoprotein chains of PBS core) was solvated in a truncated octahedron box of ∼14-nm diameter, ensuring at least 4-nm spacing between periodic images of the protein complex. Na^+^ ions were added to neutralize the system. The protein was described with the AMBER ff14SB force field (*50*), whereas, for CAN, we used our previously developed force field (*20*) and the general Amber force field (GAFF) for PCBs (*51*). Water was described with the TIP3P model. The entire system was minimized subject to restraints of 4 kcal mol^−1^ Å^−1^ on all non-solvent non-hydrogen atoms. Then, the system was heated gradually to 300 K in 20 ps, with the same restraints in the NVT ensemble. Last, the box was equilibrated through a 1-ns constant temperature and pressure simulation using the Monte Carlo barostat implemented in Amber, keeping the restraints. All simulations were run with the Langevin thermostat, a time step of 2 fs, and the SHAKE algorithm. Periodic particle-mesh Ewald (PME) electrostatics was used with a short-range cutoff of 1 nm. The restMD production was run for 40 ns subject to restraints of 4 kcal mol^−1^ Å^−1^ on the backbone of the proteins. Only the OCP linker residues (169 to 179) were allowed to move freely. The internal geometry of the CAN molecule was frozen to the previously QM/MM optimized geometry, whereas the overall position of CAN was allowed to fluctuate. For the geometry optimization of CAN, we used density functional theory (DFT) at the B3LYP/6-31G(d) level. This strategy allows us to sample the configurations of the system by allowing full freedom of movement to the parts that are not resolved in the EM map while restraining the parts where the EM density gives full confidence on the atomic positions. At the same time, we always use the DFT optimized geometry for the CAN, so that bond lengths and angles are fully relaxed.

### Excited-state QM/MM calculations

Excited states of CAN were determined with a semiempirical configuration interaction (CI) method with parameters optimized specifically for carotenoids (*21*). Excited-state calculations on the bilins were performed using time-dependent DFT at the CAM-B3LYP/6-31G(d) level. For calculations in OCP-PBS, we used an electrostatic embedding QM/MM scheme including point charges of the protein, other cofactors, water molecules, and ions. Transition charges were obtained from the transition electrostatic potential, as obtained from QM/MM calculations, in accordance with the TrEsp method (*25*). The coupling between pigments A and B was calculated as

$$V_{\text{AB}}=\sum_{i}^{A}‍\sum_{j}^{B}‍\frac{q_{i}q_{j}}{r_{\mathrm{ij}}}$$

where *q_i_* and *q_j_* are the transition charges on atoms *i* of pigment A and atom *j* of pigment B, respectively, and *r_ij_* is the distance between them.

The effect of protein residues on the excitation energy and TDM of CAN’s excited states was determined as follows: For each considered residue, we repeated all the QM/MM calculations after setting to zero all the charges of its side chain. The resulting TDM (μ) was then compared to the reference QM/MM calculation that includes all charges (μ^ref^), and the logarithm of the fold change log(μ/μ^ref^) was calculated for each snapshot of the MD. Note that this analysis neglects any structural changes that may arise from removing the residue.

### Energy transfer modeling

Simulation of the EET dynamics has been performed essentially as described in (*4*). We used Förster theory to compute the pairwise energy transfer rates, from couplings (*V*) and overlaps (*J*) of normalized absorption and emission spectra, using equation $k_{\text{AB}}=1.18\;V_{\text{AB}}^{2}\;J$ (*52*). As the full absorption profile of the CAN S_0_-S_1_ transition is not known, we used the emission line shape of another keto-carotenoid, peridinin (*53*), assuming a mirror image relationship between absorption and emission line shapes (*54*). A detailed balance condition was applied to account for uphill energy transfer rates. The simulation was then run using the stochastic approach, using the Gillespie algorithm (*55*, *56*). Each simulation run started from a randomly selected rod PC bilin, and, for each run, the carotenoid parameters were independently sampled.
