# Supplementary material for: Structural and quantum chemical basis for OCP-mediated quenching of phycobilisomes
Source: Sci Adv. 2024 Apr 5;10(14):eadk7535. doi: 10.1126/sciadv.adk7535 (PMC10997198; doi:10.1126/sciadv.adk7535)
Supplement: Supplementary file 1 — Figs. S1 to S11 Table S1 Legends for movies S1 to S3 [file sciadv.adk7535_sm.pdf]

Science Advances
AAAS

Supplementary Materials for

Structural and quantum chemical basis for OCP-mediated quenching of phycobilisomes

Paul V. Sauer *et al.*

Corresponding author: Paul V. Sauer, psauer@berkeley.edu; Lorenzo Cupellini, lorenzo.cupellini@unipi.it; Benedetta Mennucci, benedetta.mennucci@unipi.it

Sci. Adv. **10**, eadk7535 (2024)
DOI: 10.1126/sciadv.adk7535

The PDF file includes:

Figs. S1 to S11
Table S1
Legends for movies S1 to S3

Other Supplementary Material for this manuscript includes the following:

Movies S1 to S3

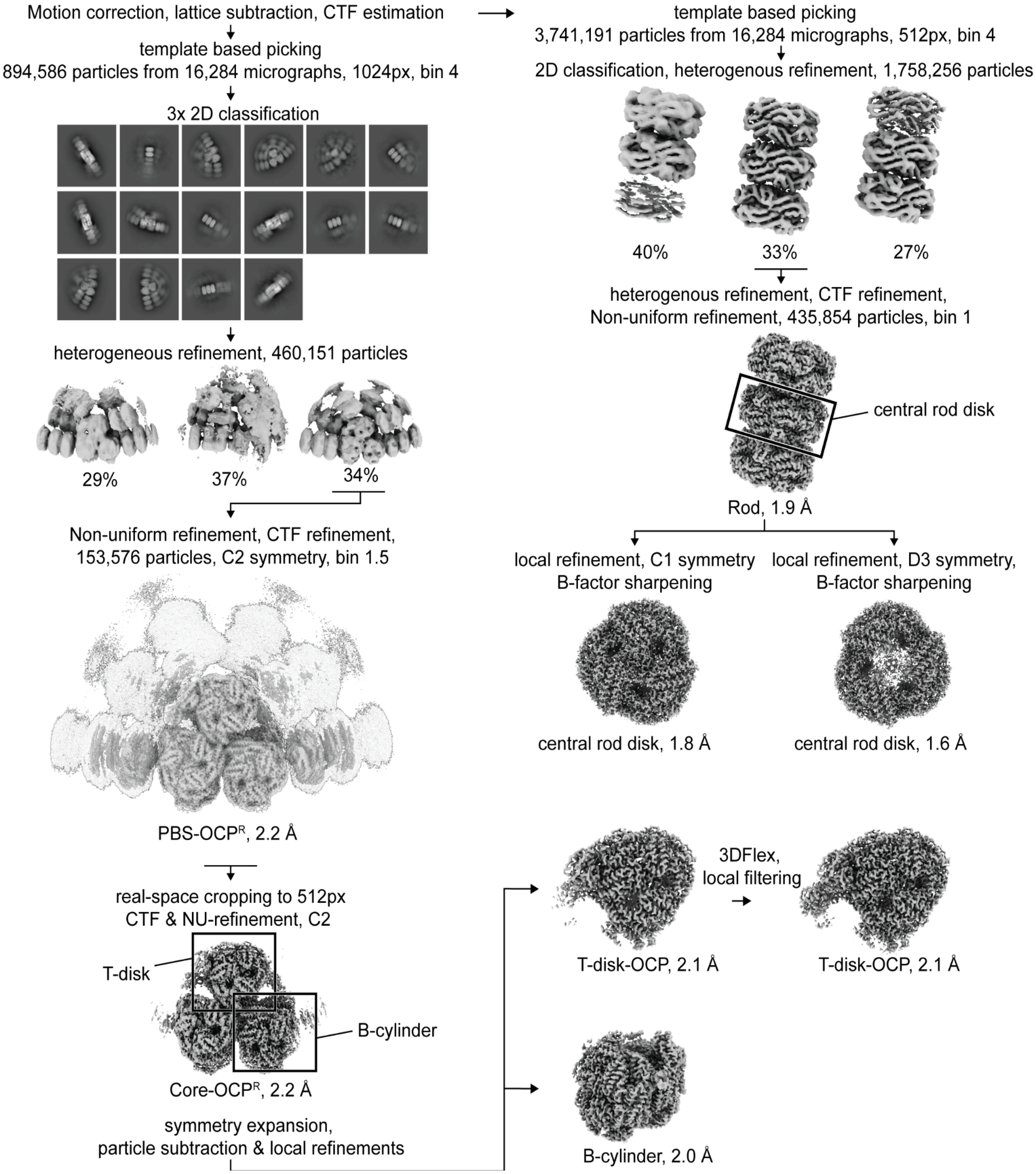

Figure S1: **Cryo-EM processing workflow.** Holo-OCP-PBS particles and the rods were processed independently.

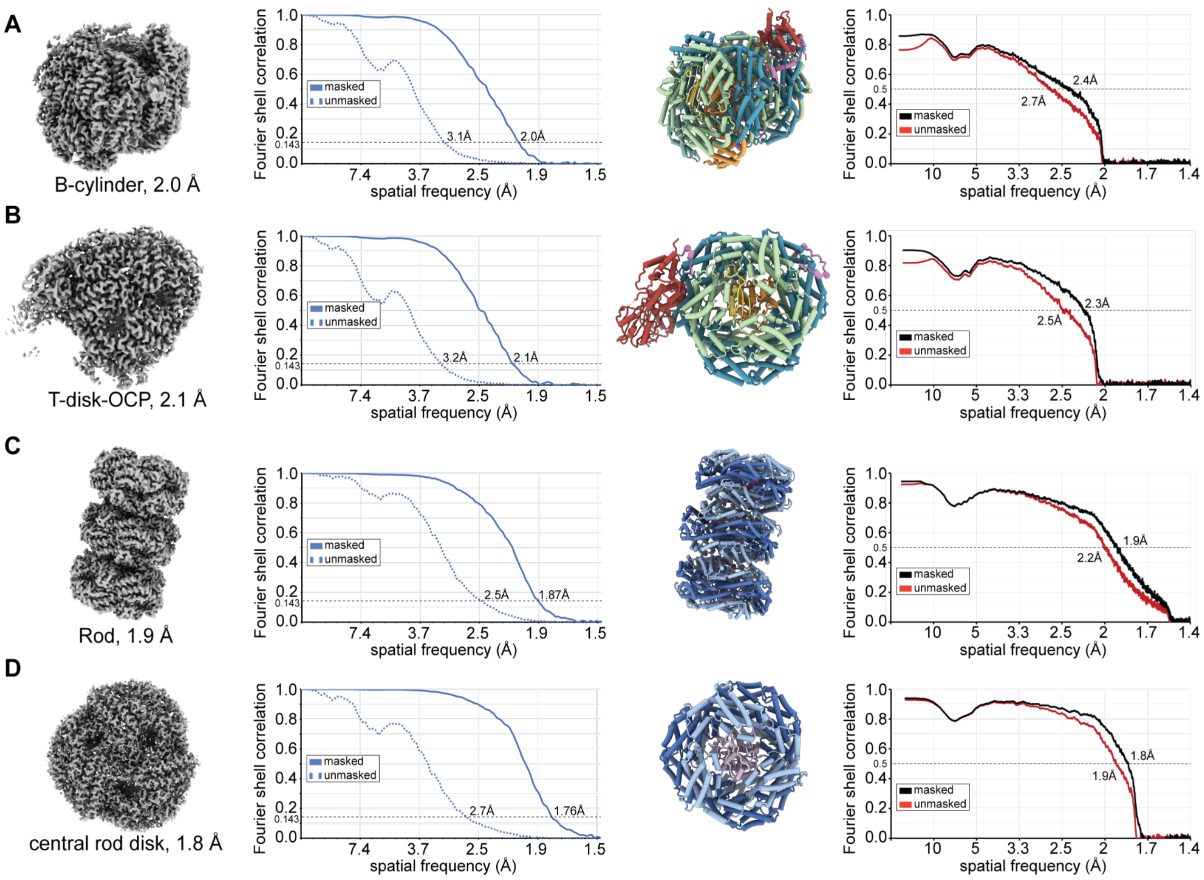

Figure S2: **Fourier shell correlation curves and model validation.** From left to right, final cryo-EM reconstructions (identical to Figure S1), their corresponding Fourier shell correlation curves, their atomic models and the map-to-model validation plots, shown for the B-cylinder-OCP (A), the T-disk-OCP (B), the rod (C), and the central rod disk (D). The entire OCP-PBS model can be put together from the partial models (A)-(C) and the cryo-EM map of the complete complex.

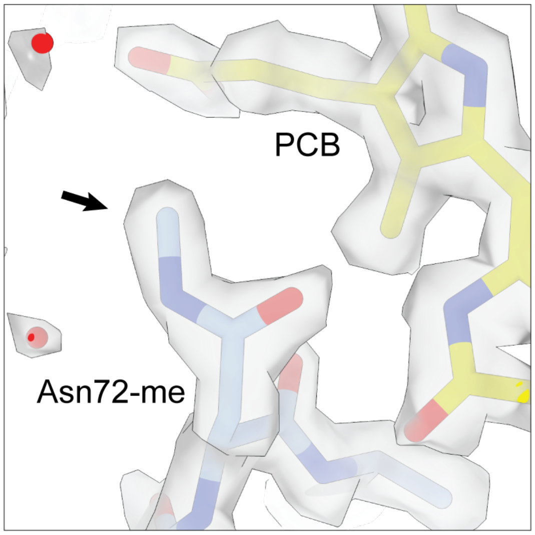

Figure S3: N4-methylasparagine in position 72 of CpcB, presumably the result of post translational modification, adjacent to a phycocyanobilin (PCB). Arrow indicates additional methyl group.

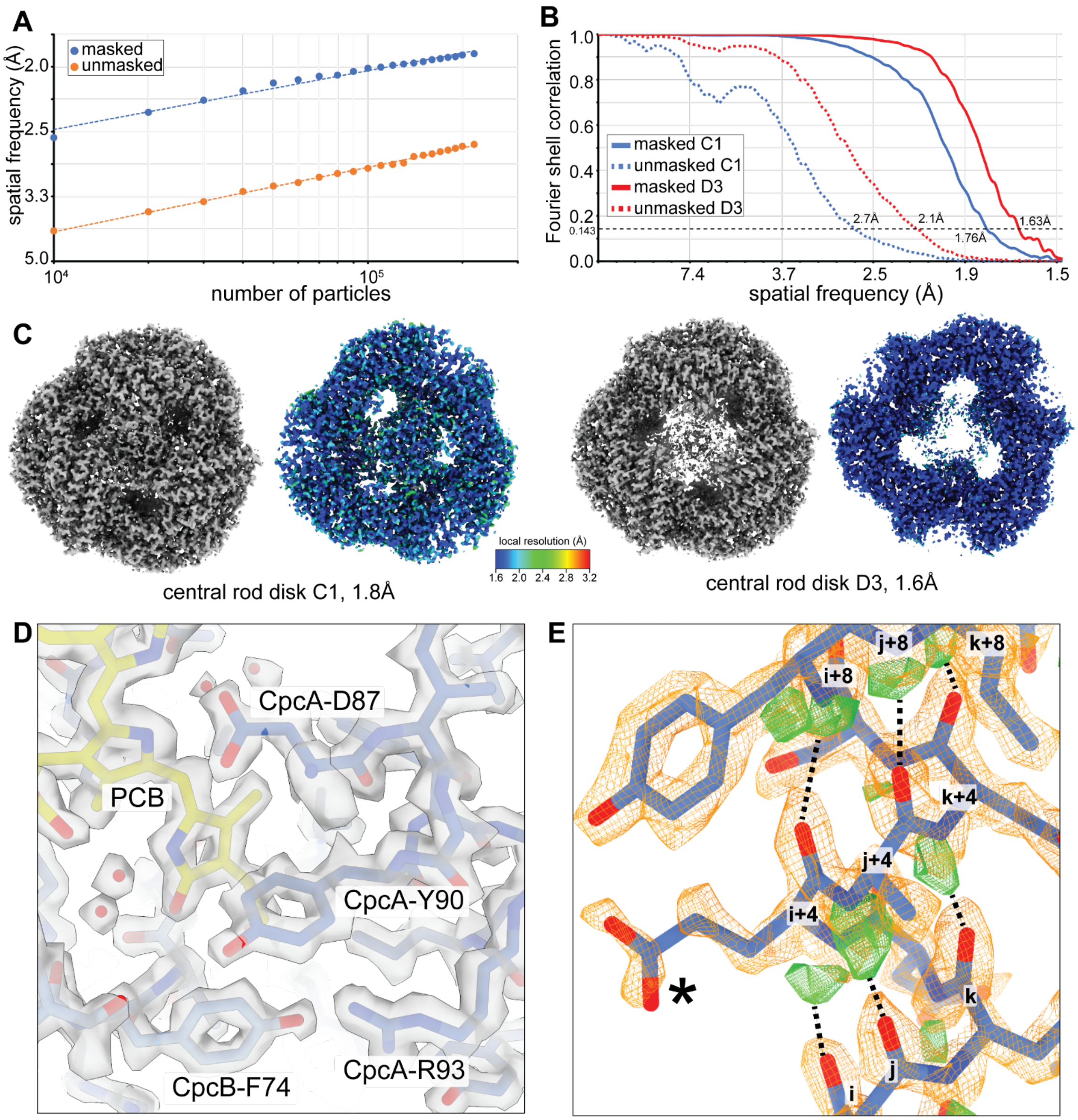

Figure S4: **High resolution details of the central rod segment.** (A) ResLog plot of the full rod showing that attainable resolution is limited by particle numbers. (B) Fourier shell correlation curves from local reconstructions of the central rod disk, using either C1 or D3 symmetry. (C) Cryo-EM maps of the central rod disk either using C1 symmetry (left) or D3 symmetry (right). Next to each map a slice of the local resolution map is presented, showing the resolution in the interior. (D) High resolution map detail of the 1.6 Å D3 symmetry rod disk map. Water molecules and holes in aromatic residues are clearly visible. (E) FO map of an alpha helix from the C1 symmetrical central rod disk (orange mesh). The F_O-F_C map (green mesh) shows differential density indicating the potential position of hydrogen atoms. The hydrogen positions are in agreement with the hydrogen bonding patterns in alpha helices, which bridge the carbonyl moiety of residue i with the peptide bond of residue $i+4$. Potential hydrogen bonds are indicated with dotted lines. Asterisk marks a terminal carboxyl group of a glutamate residue that has disconnected density from the backbone, presumably a result of beam induced decarboxylation. F_O-F_C maps have been calculated according to (*48*).

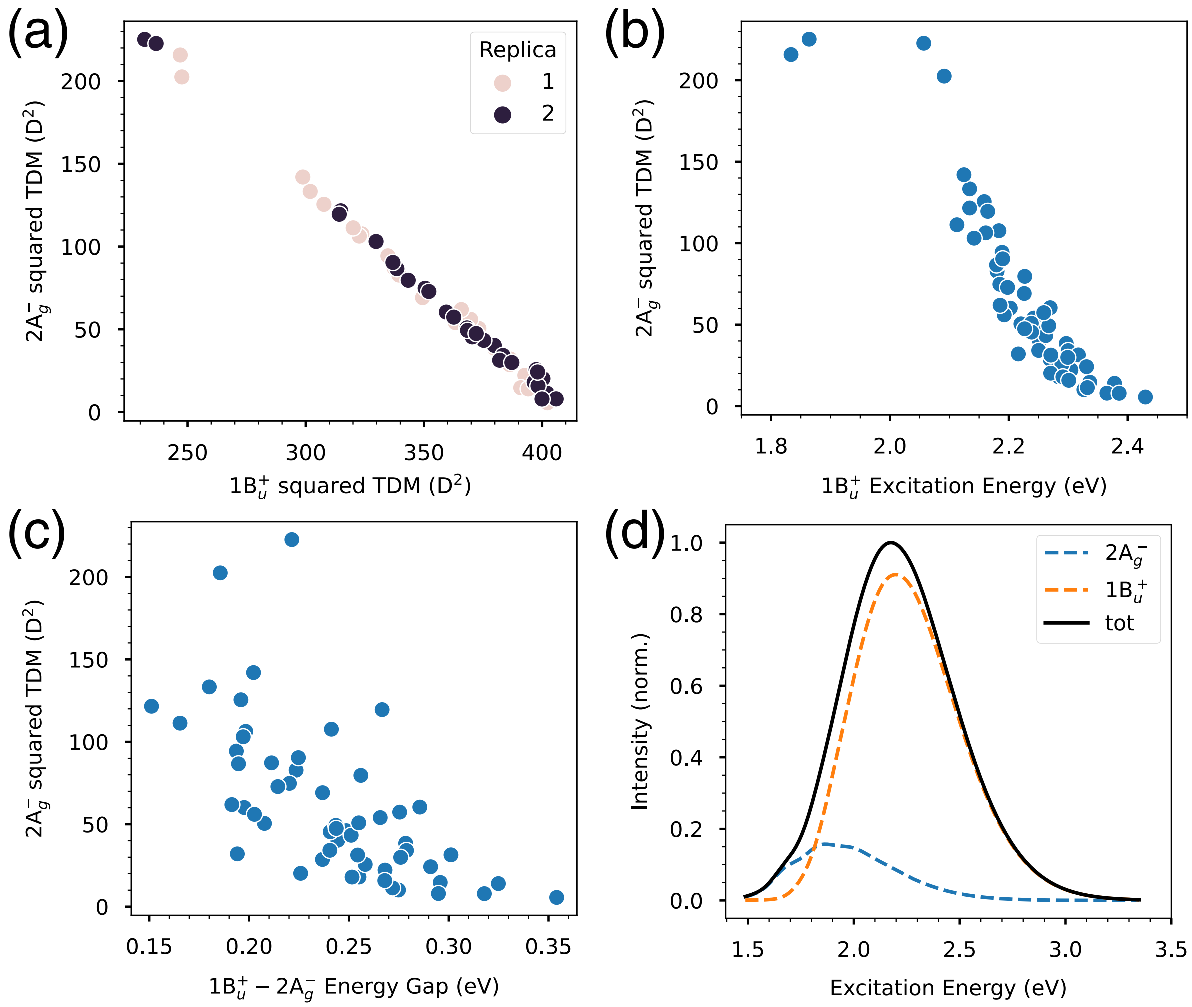

Figure S5: **Demonstration of S_1-S_2 mixing for CAN in OCP-PBS.** (a) Correlation between the squared TDMs of S_0-S_1 ($2A_g^-$) and S_0-S_2 ($1B_u^+$) transitions. Points of different colors represent structures extracted from two different MD trajectories (replicas). (b) Correlation between the squared TDM of the S_0-S_1 ($2A_g^-$) transition and the S_0-S_2 ($1B_u^+$) excitation energy. (c) correlation between the squared TDM of the S_0-S_1 transition and the S_2-S_1 energy gap. (d) Spectrum calculated by convoluting the S_1 and S_2 contributions with the vibronic lineshape of the two transitions.

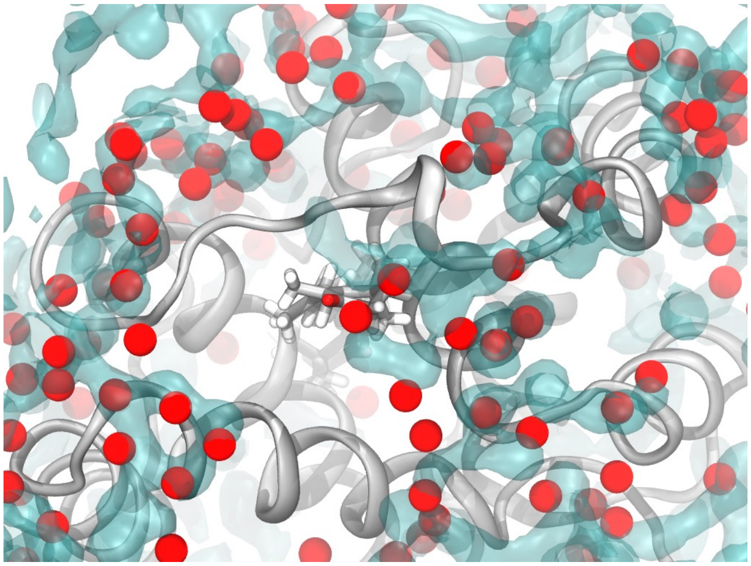

Figure S6: **Water occupancy at the OCP-PBS interface during the restMD simulations.** The OCP NTD is shown (cartoon representation) along with CAN (licorice). The red spheres represent water molecules determined in the Cryo-EM model, whereas the cyan surface shows the regions of high number density ($> 0.04/\text{Å}^3$) of water oxygen atoms. The water density was obtained throughout the restMD trajectories of OCP-PBS

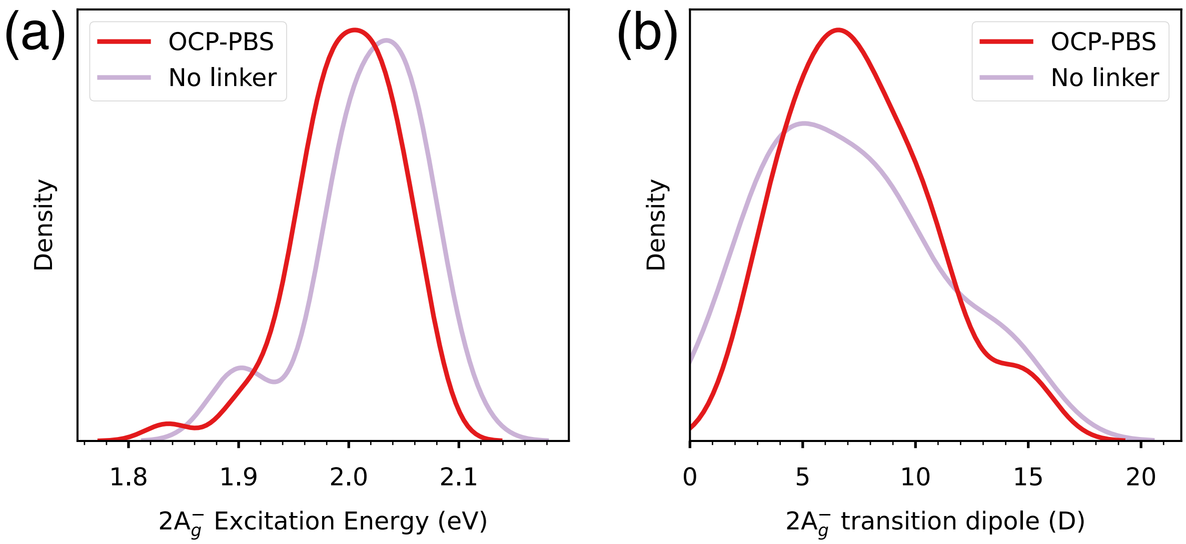

Figure S7: **Effect of the linker.** Distributions of (a) S_0-S_1 ($2A_g^-$) transition energies and (b) S_0-S_1 ($2A_g^-$) transition dipole moments (right) in the OCP-PBS model and without the linker. Data correspond to two pairs of completely independent MD replicas.

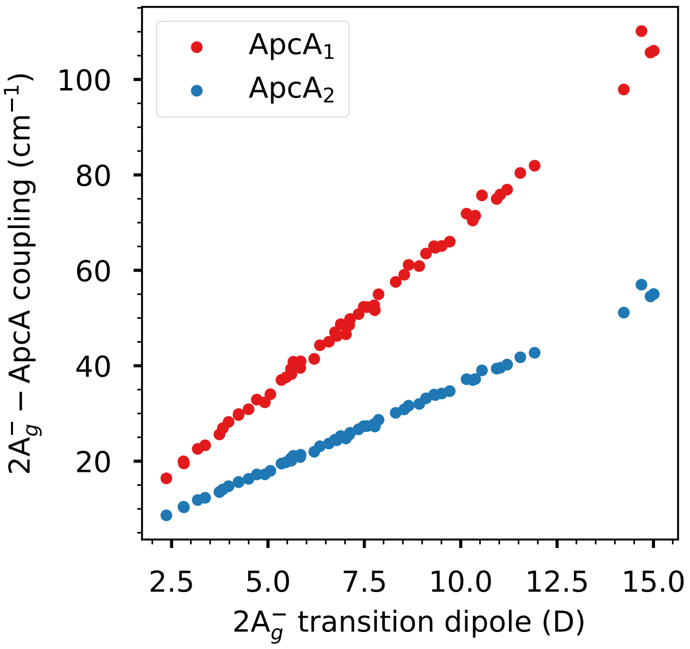

Figure S8: Correlation between the S_0-S_1 ($2A_g^-$) TDM of CAN and the coupling to ApcA1/2.

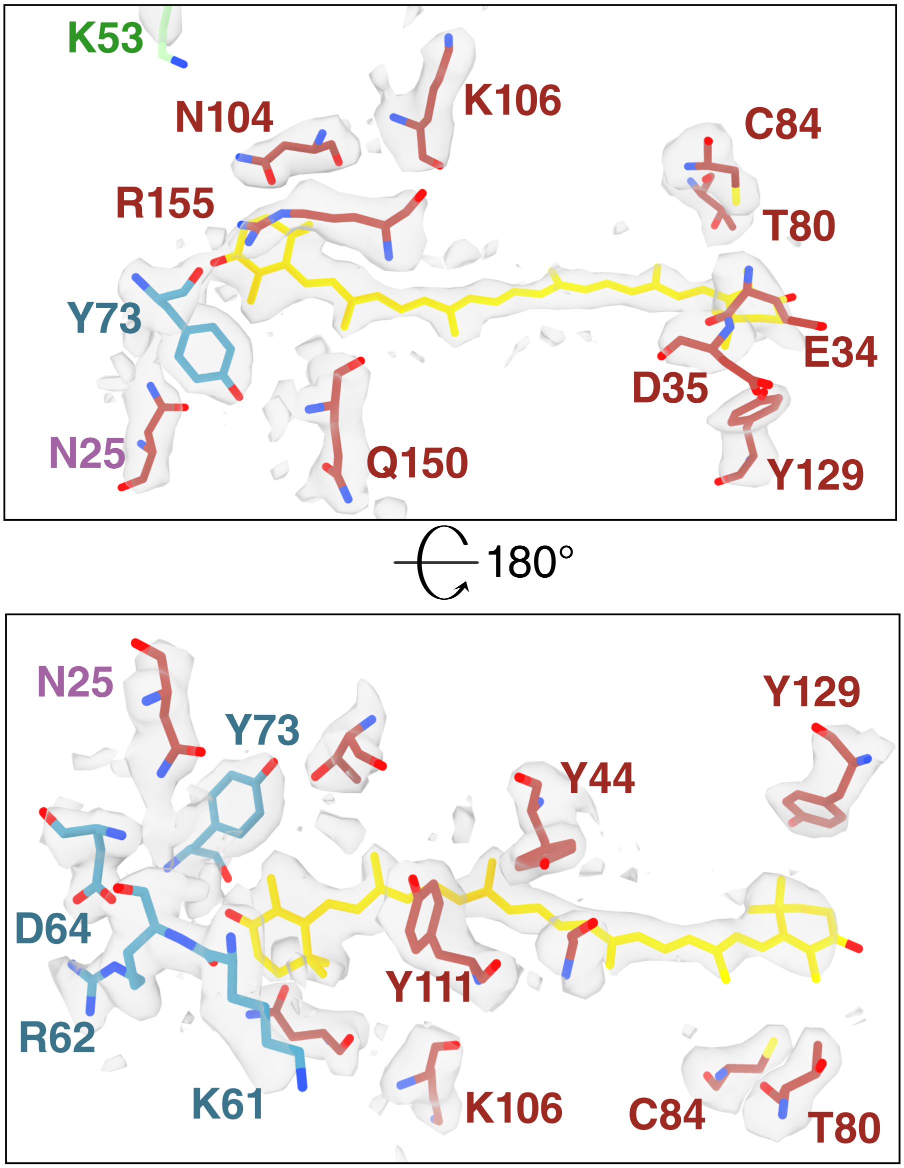

Figure S9: **OCP and PBS residues surrounding the CAN in the average MD structure.** The residues are superimposed to the experimental cryo-EM map of this work (A,C) and of the earlier work ref. (4) (B,D). The closest polar or charged residues to CAN are shown.

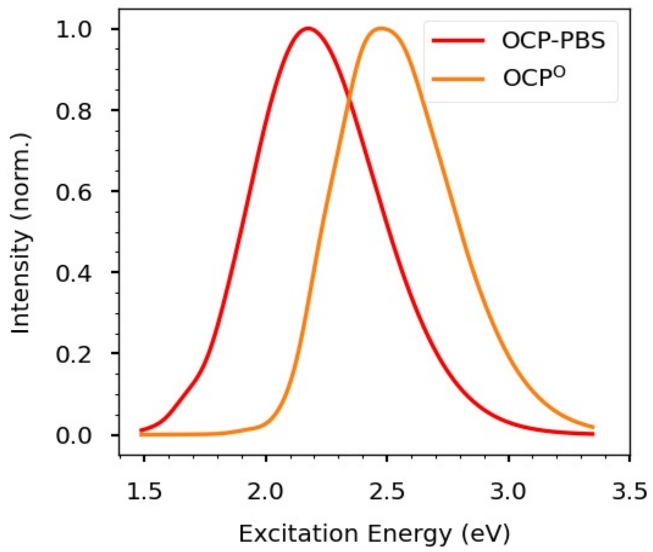

Figure S10: **Comparison between the computed absorption spectra of OCP^O and OCP-PBS.** Both spectra have been calculated by convoluting the S_1 and S_2 contributions with the vibronic lineshape of the two transitions. The contribution from the S_1 state is completely negligible for OCP^O, whereas for OCP-PBS the two contributions are shown in Figure S5d.

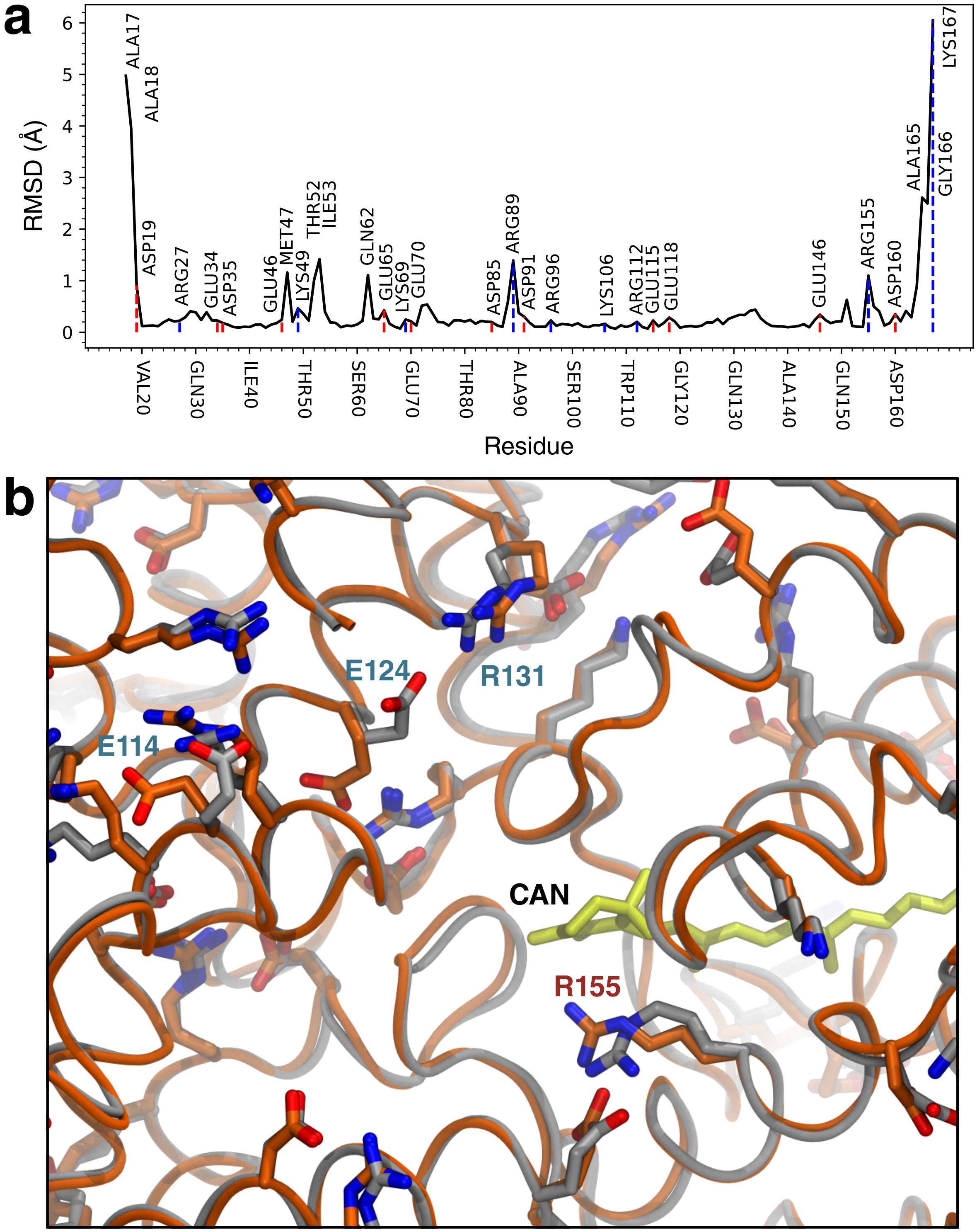

Figure S11: **Differences between previous and current structure.** (a) Per-residue minimum RMSD between frames of the restMD simulations on the two structures. Charged residues and residues with more than 1 Å RMSD are labeled. (b) Differences between representative restMD samples of the low resolution (grey) and high resolution (orange) structures at the OCP-PBS interface. Only charged residues are shown, and residues showing significant differences are labeled (red labels refer to the OCP sequence, whereas cyan labels refer to the ApcA sequence).

Table S1: **Cryo-EM data collection and refinement parameters**

**Title**	**Holo OCP-PBS**	**T-disk OCP**	**B-cylinder**	**rod**	**central rod disk C1**	**central rod disk D3**
**EMD**	41463	41475	41434	41585	41435	41436
**PDB**		8TPJ	8TO2	8TRO	8TO5	
	**Data collection and processing**					
Magnification	192 000 x					
Voltage (kV)	300					
Electron exposure (e–/Å^2)	40					
Defocus range (μm)	0.4 - 1.2					
Pixel size (Å)	0.727					
Symmetry imposed	C2			C1		D3
Initial particle images (no.)	894 586			3 741 191		
Final particle images (no.)	153 576			435 854		
Map resolution (Å) FSC threshold 0.143	2.2	2.1	2.0	1.9	1.87	1.63
Map resolution range (Å)	2-12	1.8-12	1.8-4	1.8 - 2	1.7 - 2	1.5-1.8
	**Refinement**					
Initial model used (PDB code)		7SCC	7SCB	7SCA	7SCA	
Model resolution (Å) FSC threshold 0.5		2.2	2.4	1.9	1.8	
	Model composition					
Non-hydrogen atoms		39096	74379	110459	37853	
Protein residues		2463	4737	6835	2295	
Ligands		13	25	54	18	
	B factors (Å^2)					
Protein		22.9	19.3	15.3	25.5	
Ligand		20.4	16.0	14.0	24.6	
	R.m.s. deviations					
Bond lengths (Å) (# >4σ))		0.006 (0)	0.004 (0)	0.005 (0)	0.003 (0)	
Bond angles (°) (# >4σ))		0.654 (7)	0.644 (19)	0.647(4)	0.619 (4)	
	Validation					
MolProbity score		1.20	1.31	1.21	1.04	
Clashscore		4.22	5.71	4.26	2.56	
Poor rotamers (%)		0.00	0.00	0.38	0.23	
	Ramachandran plot					
Favored (%)		98.59	98.14	98.45	99.29	
Allowed (%)		1.41	1.78	1.55	0.71	
Disallowed (%)		0.00	0.09	0.00	0.00	

Movie S1

Representative motion of the holo OCP-PBS complex, determined with 3DVA (*13*). The rods move 'up' and 'down' relative to the core as rigid bodies. The position of OCP in the complex is indicated. Related to Fig. 2A.

Movie S2

Representative motion of the OCP-PBS core, determined with 3DVA (*13*). The core itself displays a rocking and twisting motion. The ApcA/B hexamer that is absent in a subset of particles is indicated. Continuous flexibility causes the OCP density to appear fragmented. Related to Fig. 2B.

Movie S3

Representative motion of the T-disk bound to OCP, determined with 3DVA (*13*). Relative to the T-disk, the OCP-CTD is flexible. It is connected to the OCP-NTD via a flexible linker. The CAN is in close proximity (indicated). Related to Fig. 2C.
